# Are dietary interventions with a behaviour change theoretical framework effective in changing dietary patterns? A systematic review

**DOI:** 10.1186/s12889-020-09985-8

**Published:** 2020-12-03

**Authors:** Deirdre Timlin, Jacqueline M. McCormack, Maeve Kerr, Laura Keaver, Ellen E. A. Simpson

**Affiliations:** 1 School of Psychology, Ulster University, Coleraine, UK; 2 Faculty of Science, Sligo Institute of Technology, Sligo, UK; 3 Nutrition Innovation Centre for Food and Health (NICHE), Ulster University, Coleraine, UK; 4 Psychology Research Institute, Ulster University, Coleraine, UK

**Keywords:** Psychological theory, whole dietary patterns, theory coding scheme

## Abstract

**Background:**

The term ‘whole dietary pattern’ can be defined as the quantity, frequency, variety and combination of different foods and drinks typically consumed and a growing body of research supports the role of whole dietary patterns in influencing the risk of non-communicable diseases. For example, the ‘Mediterranean diet’, which compared to the typical Western diet is rich in fruits and vegetables, whole grains, and oily fish, is associated with reduced risk of cardiovascular disease and cancer. Social Cognition Models provide a basis for understanding the determinants of behaviour and are made up of behavioural constructs that interventions target to change dietary behaviour. The aim of this systematic review was to provide a comprehensive assessment of the effectiveness and use of psychological theory in dietary interventions that promote a whole dietary pattern.

**Methods:**

We undertook a systematic review using the Preferred Reporting Items for Systematic Reviews and Meta-Analysis to synthesize quantitative research studies found in Embase, Medline, PsycInfo, CINAHL and Web of Science. The studies included were randomised and non-randomised trials published in English, involving the implementation of a whole dietary pattern using a Social Cognition Model to facilitate this. Two independent reviewers searched the articles and extracted data from the articles. The quality of the articles was evaluated using Black and Down quality checklist and Theory Coding Scheme.

**Results:**

Nine intervention studies met the criteria for inclusion. Data from studies reporting on individual food group scores indicated that dietary scores improved for at least one food group. Overall, studies reported a moderate application of the theory coding scheme, with poor reporting on fidelity.

**Conclusion:**

To our knowledge, this is the first review to investigate psychological theory driven interventions to promote whole dietary patterns. This review found mixed results for the effectiveness of using psychological theory to promote whole dietary pattern consumption. However, the studies in this review scored mostly moderate on the theory coding scheme suggesting studies are not rigorously applying theory to intervention design. Few studies reported high on treatment fidelity, therefore, translation of research interventions into practice may further impact on effectiveness of intervention. Further research is needed to identify which behaviour change theory and techniques are most salient in dietary interventions.

**Supplementary Information:**

The online version contains supplementary material available at 10.1186/s12889-020-09985-8.

## Background

Major non communicable diseases (NCD) include, heart disease, stroke, cancer, diabetes, and chronic respiratory disease, and are estimated to represent 41 million deaths per annum globally [1]. According to the World Health Organisation (WHO, 2016) [1], a number of preventable risk factors underlie many NCD’s and are the leading cause of death and disability globally regardless of economic status with one of the main risk factors considered to be poor diet attributable to 11 million deaths globally in 2017 [2]. Previously, the focus of research has been on single nutrients or single food groups, as the main disease states were due to nutritional deficiencies. However, the burden of disease has switched [2] to cancer, diabetes, and cardiovascular disease, due to demographic and epidemiological transitions, which are now the leading causes of death globally. This was partly due to a shift in the food environment, with people consuming more high-carbohydrate, low fat diets, which in turn lead people to consume more refined carbohydrates and refined sugar, which increases the risk of cardiometabolic diseases [3]. The dietary determinants of diseases such as cancer and diabetes are different from those of undernutrition and nutrient deficiency states [4]. Non-communicable diseases have multiple interacting dietary determinants consisting of either excess or insufficient intake, which cumulatively affect disease over time [5]. Therefore, research has gone beyond the single nutrient approach and focused on whole dietary patterns, which may be more beneficial to health due to the synergism between nutrients and food groups [6].

Improving dietary quality is not easily achieved. Healthy eating patterns revolve around regular consumption of a variety of foods from key food groups including cereal and cereal products, fruits and vegetables, meat and non-meat alternatives and dairy/non-dairy alternatives with the aim of optimizing nutrient intakes conducive to reducing the risk of chronic illness [7]. Globally between 1990 and 2010, consumption of healthy foods has increased, however, the consumption of unhealthy foods had increased to a greater extent [8]. As opposed to a “healthy” dietary pattern, which can be nutrient based or only focus on certain aspects of a diet, for the purpose of this review, a whole dietary pattern is defined as the quantities, proportions, variety or combination of different foods in relation to the 5 foods groups of the Eatwell Guide, UK [9] and the MyPlate, USA [10] (fruit & vegetables, carbohydrates/grains, protein, fats & sugar, dairy products), or an established healthy eating pattern such as the Mediterranean diet [11].

It is clear, that interventions to promote adherence to a healthy dietary pattern are warranted. There is an array of research examining and evaluating the effectiveness of dietary interventions on chronic illnesses. There is some evidence in the literature to suggest, that the reporting of psychological theory use in behaviour change intervention development is associated with larger intervention effects [12]. Using psychological theory to design behaviour change interventions, provides a framework to accumulate evidence, test hypothesis, identify specific constructs that may influence behaviour and suggest which behaviour change techniques should be used in behavioural interventions [13].

Social Cognition Models (SCMs) (e.g. Theory of Planned Behaviour (TPB)) [14] are the most commonly used theories within the field of health psychology and behaviour change [15]. SCMs are useful for explaining, predicting, and understanding dietary behaviours, and in the design of dietary interventions to promote dietary change [16]. However, while SCMs has been used to predict dietary patterns [17, 18], there is less evidence in the literature examining the effectiveness of interventions that use SCMs to promote whole dietary patterns, such as the Mediterranean [11], MIND [19], and DASH [20] diets. However, reviews in the literature show mixed results for the effectiveness of theory based dietary interventions. One meta-analysis found no association between dietary intervention effectiveness and theory use [21], while another meta-analysis on theory-based fruit and vegetable intervention among children, found that after considering quality of studies, theory was associated with vegetable consumption only [22]. Furthermore, a previous review indicated that theory-based interventions were less effective than non-theory-based interventions [23]. However, such research is held back by limitations in the extent to which interventions report on theory use, and insufficient descriptions of intervention content [24].

Some studies have been shown not to extensively use psychological theory in developing interventions [25]. One way to examine how theory has been applied to interventions is by applying the 19-item theory coding scheme (TCS) [26]. This scheme specifies whether theory is mentioned, whether theoretical constructs are targeted or measured, if theory was used to select recipients or to tailor the intervention and if theory was tested or refined. The TCS is a reliable tool to describe theory-based interventions; to inform evidence synthesis within reviews and has been used widely in systematic reviews to assess the effectiveness of theory and intervention effectiveness.

To advance behavioural research, improvement in methodologies are needed, with treatment fidelity proposed as a key area for improvement. Treatment fidelity refers to the processes used to ensure intervention components are delivered as intended [27]. To make valid interpretations regarding the efficacy of a behavioural intervention, it is important to provide details of treatment fidelity, which provides insights into the gap between theory and practice. To provide this information, specification of the intervention program is required. According to Bellg et al. [28], five domains to assess, monitor or enhance treatment fidelity have been identified by, as part of The National Institute of Health (NIH) and Behaviour Change Consortium (BCC), which are: (1) design of study, (2) training providers (3) delivery of treatment (4) receipt of treatment (5) enactment of treatment skills.

Previous systematic reviews have assessed the effectiveness of behavioural interventions on fruit and vegetable consumption [29], reduce sugar intake [30], or only reporting on dietary behaviours using one SCM, such as the Social Cognitive Theory [31]. One systematic review [31] aimed to identify effective dietary interventions for older people. However, this review examined both whole dietary patterns and single food groups such as fruit and vegetables. Furthermore, while this review reported the delivery of educational sessions, no theory was mentioned, or theoretical constructs reported. To our knowledge, the current review is the first to assess the effectiveness of SCMs in dietary interventions that use a “whole dietary pattern”. Therefore, the aim of this systematic review was to provide a comprehensive and systematic assessment of the effectiveness and use of SCMs in dietary interventions that promote “whole dietary patterns” in adults.

### Objectives

- To describe the extent of psychological theory in the design and implementation of dietary interventions to promote whole dietary patterns
- To evaluate the implementation of psychological theory in the design of dietary interventions to promote whole dietary patterns
- To determine the effectiveness of psychological theory based dietary interventions
- To explore the extent to which the fidelity of the intervention is monitored in these studies.
- To provide recommendations for future research to promote whole dietary patterns

## Methods

The Preferred Reporting Items for Systematic Reviews and Meta-Analysis (PRISMA) (see supplementary data file 1) [32] was used to inform the design, conduct and reporting of this systematic review. No ethical approval was sought as only secondary analysis of existing datasets were involved in the study. The study protocol was registered with PROSPERO, the International Prospective Register of Systematic Reviews (crd.york.ac.uk/prospero/index.aspIdentifier; CRD42017057366).

### Selection criteria

In accordance with PRISMA, the PICOs (population, intervention, comparison, outcome, and study design) approach were used to formulate the selection criteria. (see Table 1).

**Table 1:** Description of Population, Intervention, Comparison, Outcome and Study Design for Included Studies (PICOS)

Parameter	Description
Population	All adults aged 18 years and over. Studies where participants were drawn from a population with a psychiatric condition such as an eating disorder were excluded.
Intervention	Diet: for the purpose of this review, an intervention involving a “whole dietary pattern” such as the Mediterranean diet [11] Dash diet [18] MIND diet [17] or foods analysed from 4 out of 5 of the food groups (protein, grains/carbohydrates, oil and fats, dairy, fruit/vegetable. Studies were not included where the dietary behaviour was a de facto medical treatment e.g. gluten free diet, also single food group and nutrients diet studies such as, fruit and vegetable, or omega 3 were excluded, as these don’t from a “whole dietary” pattern. Theoretical model: Studies were included that used a theoretical framework to deliver their intervention. Theoretical models such as the Health Belief Model (HBM) [84] Stages of Change Model [85] and Health Action Process Approach (HAPA) [86] were included.
Comparison	Usual diet, information booklet.
Outcome	Improved diet quality, increased adherence to diet.
Study design	Randomised controlled trials and non-randomised controlled trials published in English.

Inclusion criteria: 1) study population: all adults aged 18 years or over; 2) study intervention: an intervention involving a “whole dietary pattern” such as the Mediterranean diet [11] Dash diet [18] and MIND diet [17] or foods analysed from at least 4 out of 5 of the food groups identified by the Eatwell Guide, UK (protein, grains/carbohydrates, oil and fats, dairy, fruit/vegetable; 3) psychological theory: studies were included that used a social cognition model to design their intervention (e.g. TPB); 4) study design: randomised controlled trials and, including single arm studies, and pilot studies published in English.

Exclusion criteria: 1) study population; studies targeting a population under 18 years old were excluded; 2) study intervention: studies were not included where the dietary behaviour was a de facto medical treatment e.g. gluten free diet. Also, studies analysing data from only single food group and nutrients, such as, fruit and vegetable, or omega 3 were excluded, as these do not constitute a “whole dietary pattern”; 3) psychological theory: studies that do not mention or report on a social cognition model were excluded; 4) study design: studies that were not interventions, such as qualitative or cross-sectional studies were excluded from this review. (see Table 1).

### Search strategy and study identification

Literature searches were conducted by (DT) between April 2019 and January 2020 using the following databases: EMBASE (1974–2020), Medline (1974–2020), PsycInfo (1974–2020), CINAHL (1937–1-2020) and Web of Science (1950–2020). ProQuest Dissertations & Thesis was reviewed to locate unpublished studies and, reference lists of the selected studies for inclusion were searched manually. The following search terms were used in different combinations. Theoretical framework, behaviour change theory, Theory of Planned Behaviour, Theory of Reasoned Action, Health Belief Model, Self-determination Theory, Stages of Change Model, Health Action Process Approach, COM-B model, Social Cognitive Theory, Control Theory, Self-Efficacy Theory, Social Ecological Model, healthy eating, dietary intervention, dietary patterns, healthy eating, whole diets, Mediterranean, DASH and MIND diet were also chosen as search terms as these are “whole dietary patterns”, they do not eliminate any food group and promote a healthy lifestyle [7]. The studies were screened by the titles and abstracts. Studies that did not meet the inclusion criteria were excluded. Two researchers (DT&ES) reviewed the abstracts independently that were ambiguous for inclusion. Any disagreements were resolved through discussion with a third researcher (JM).

### Data extraction

The following information was extracted from each study: author, design, country, quality score, participants characteristics, intervention, control, dietary pattern, theoretical model, outcome measures, main findings (Table 2).

**Table 2:** Data Extraction: Description of Study Characteristics in Theory Based Dietary Interventions Promoting Adherence to a Whole Dietary Pattern

Author Design Country	Theoretical Model	Participants	Intervention	Control	Dietary Pattern	Primary outcome	Main findings
Abood, D.A et al. [33] 2003 RCT USA	Health Belief Model	53 participants in the study. *N* = 28 intervention, mean age 34, 96% women. *n* = 25 control mean age 38, 92% women	Pre-post-test. 8 1-h weekly education session to promote knowledge and beliefs conducive to improving positive dietary practices. INTERVENTION 1. Risk factors and prevalence rates of CVD, nutrition to reduce risk. 2. Macronutrients: food guide pyramid and sources and benefits of recommended intakes, benefits of proper nutrition, reducing barriers to increase probability of dietary changes, 3. Macronutrients; hidden sources of fat, meal and fat alternatives, benefits of fat reduction and reduction of barriers to taking such action. 4. Fruit and veg: Health protective role of fruit and veg, frequency and portion size, fibre, vitamins, benefits of increased intake of fruit and veg and barrier reduction to taking action. 5. Health benefits of weight control. 6. Benefits of eating meal regularly; distribution and preparation of low calorie-high nutrient recipes, ideas for removing barriers to healthy eating patterns. 7. Meal planning and food label reading. 8. Integration of all previous topics; HMB constructs to change nutrition behaviours to reduce risks and for behaviour maintenance; supplements, caffeine, soft drinks.	Usual care	Dietary behaviour (Whole dietary pattern)	Modified FFQ used by Boeckner and colleagues (1990) Questionnaire on HBM.	Following the intervention, there was a significant improvement in total fat, saturated fat. No significant effect for protein, fibre, fruit, or veg.
Manios, Y et al. [34] 2007 RCT Greece	Health Belief Model (HBM) Social Cognitive Theory (SCT)	82 women aged 55–65. Postmenopausal. Intervention *n* = 42 Control *n* = 40 Mean age 60 + − 4.8 years	Every 2 weeks in a nutrition education based on HBM and SCT over 5 months. INTERVENTION: 7 sessions based on the HBM 1. Perceived severity (What is osteoporosis) 2. Perceived susceptibility, severity, call for action (risk for osteoporosis: lifestyle choices) 3. Perceived benefits and barriers. (dietary discussion and results so far) 4. Self-efficacy, perceived barriers. Guidelines for dietary records. 5. Self-efficacy and perceived barriers. (Discussion on dietary results and changes so far) 6. Perceived benefits (Other benefits of diet) 7. Self-efficacy and perceived barriers. (Discussion of food records and barriers and benefits participants have run into)	Usual diet	Whole diet assessed by HEI. Grains, vegetable, fruit, milk, meat, total fat, saturated fat, cholesterol, sodium, total HEI	Healthy Eating Index (HEI)	Milk and Fat HEI scores were significantly improved. (p < 0.001). Significant decrease in grains (0.041) and total HEI (*P* = 0.003). Decrease in total fat was more the IG than the CG (*P* = 0.050) and also the increase in protein was more significant for the IG than the CG (*p* < 0.001) No improvement in fruit, vegetables, saturated fat
Petrogianni, M et al. [35] 2013 RCT Greece	Health Belief Model and Social Cognitive Theory	108 hypercholesterolaemia adults 40–60 years.	Randomised into 2 interventions and one control. Intervention included 7 1-h counselling and dietary lifestyle sessions held biweekly and based on HBM and SCT. 1. Perceived severity and susceptibility; cues to action (what is CVD) 2. Perceived benefits and barriers; call for action; self-monitoring; self-efficacy, (Epidemiology of CVD and ways to reduce risk factors.) 3. Perceived benefits; self-efficacy; call for action; sell-monitoring. (meal planning, setting goals) 4. Perceived benefits; self-efficacy; call for action; self-monitoring. (Guidelines for balanced diet, focus on lipids and dietary fatty acids, fasting, setting goals. 5. Perceived barriers; self-efficacy; call for action; self-monitoring. (Balanced diet plan and setting goals. 6. self-efficacy; call for action; self-monitoring. (food labels, conservatives, setting goals) 7. Progress assessment; perceived barriers and benefits. (Benefits and barriers, they have run into).	Usual diet	Dietary intake information was collected with a 3-day recall (2 consecutive weekdays and 1 weekend day)	HEI-2005 score to assess diet quality.	Significant improvement on total HEI score (*P* = 0.045), milk *p* = 0.021, dark, green vegetables and legumes *p* = 0.05
McPhail, M et al. [36] 2014 RCT Australia	Health Action Process Approach. (HAPA)	87 participants attending primary care diabetes clinic with a diagnosis of T2D.	4-month intervention consisting of self-guided HAPA based workbooks in addition to 2 telephone calls to assist participants with program implementation.	Treatment as usual	Whole diet consisting of, fruit, veg, grain, meat, dairy, beverages, sodium, saturated fat and alcohol.	Diet Guidelines Index (DGI). HAPA questionnaire.	Healthy eating was not associated with HAPA variables nor did they predict healthy eating after intervention.
Miller, C.K et al. [37] 2016 RCT USA	Health Action Process Approach. (HAPA)	68 participants aged 18–65 years. Mean age 51. 14 males, 54 females.	16-week lifestyle intervention based on the HAPA. 60-min weekly lifestyle coaching sessions. • The first 8 sessions presented the intervention goals, taught information about modifying energy intake and expenditure and helped participants self-monitor. • The following 8 sessions focussed on problem solving to achieving lifestyle goals, preventing relapse, motivating sustained behavioural change. • Action plans introduced at week 9 and later review of the success of action plan.	Control group received a booklet on lifestyle changes for diabetes prevention.	Whole diet assessed by the AHEI.	Alternative Healthy Eating Index, 2010. (AHEI) HAPA questionnaire	There was a significant increase in total AHEI score and in consumption of fruit and a significant decrease in red and processed meat, trans fat and sodium (*p* < .01).
Rodriguez, M.A et al. [38] 2019 RCT USA	(TTM) Stage of change	533 adults with uncontrolled hypertension	Tailored Intervention: **TTM** based **MONTHLY** telephone counselling for 6 months tailored to stage of change. • **Pre-contemplation/contemplation stage**: Information and feedback about achieving DASH diet, imagery exercise designed to release emotions related to DASH diet, self/environmental evaluation (goals, values, consequences of non-adherence). • **Preparation stage:** Promoting autonomy and self-efficacy towards DASH diet by thinking about past successes in behaviour change • **Action stage:** Counterconditioning; substituting unhealthy foods for healthy foods, rewarding engagement with DASH diet, introducing prompts/cues for DASH adherence • **Maintenance**: Similar to Action stage with a focus on relapse prevention. • **Decisional balance:** Pros and cons of DASH diet. Each con was addressed through problem solving and each pro was further explored. **NON-Tailored intervention**: Monthly calls to address hypertension management with general information on diet, exercise, medication, sun safety, sleep hygiene, vision/hearing problems	Usual care	DASH diet	Improve adherence to DASH diet TTM stage	Significant improvement in overall DASH score. No improvement in individual food groups Tailored intervention effectively advanced participants stage of change
Peters, N.C et al. [39] 2014 RCT USA	SCT	71 healthy post-menopausal women aged between 50 and 72	**One-year Intervention: Three eating patterns** Whole food plan, The Food Power eating plan, The Flax Plus eating plan. Behaviour Intervention: • **The first 14 weeks** (**adoption stage**) each group met weekly with behavioural classes alternating with cooking classes, to motivate participants to eat according to their eating plan. • **The following 2 months (maintenance stage)** included bi-weekly behavioural sessions including food demonstrations and tastings. • **The final 6 months (maintenance stage)** involved monthly sessions reviewing progress, goal setting and action planning.	N/A	Whole dietary pattern My Pyramid	Adherence to eating pattern with monthly 24 h recall Psycho-social questionnaire	There were no changes in psychosocial factors overtime. In the whole food eating pattern, significant improvements were found in the food group, beans and meat, poultry, eggs. In the moderate fat group, significant improvements were found for fruit, vegetables, sugar.
LeBlanc, V et al. [40] 2015 Non-RCT Canada	Self-determination theory (SDT)	64 men and 59 premenopausal women aged between 25 and 50.	Non-random intervention study. 12-week nutritional program based on STD and uses a MI approach. **INTERVENTION** 3 GROUP SESSIONS 3 INDIVIDUAL SESSIONS AND 4 FOLLOW UP PHONE CALLS. 3 GROUP SESSIONS. LECTURE; EXPLAINING PRINCIPLES OF MED DIET • 3HR Med diet cooking lesson • 3-h Mediterranean potluck dinner aimed at discussing barriers met in adopting dietary recommendations since the beginning. 3 individual sessions and follow up calls. • These assessed dietary changes and to determine progressive goals with the potential and realistic strategies aimed at improving the adherence to Med Diet principles. In accordance with the SDT, basic psychological needs were supported during the intervention (autonomy, competence, and relatedness)	No control	Mediterranean diet	Med score calculated based on validate FFQ The regulation of eating scale	Changes in eating-related self-determined motivation was positively associated with changes in Med score at follow up in men only.
Schwarzer, R et al. [41] 2017 Non-RCT Italy Spain Greece	HAPA	112 participants. 47 men 65 women Mean age 42 range 18–65 years.	Pilot intervention study. Single arm online intervention. The online platform delivered a lifestyle intervention that implemented theory-based behaviour change components based on the HAPA. INTERVENTION It is unclear how long the intervention was, this author used intervention mapping of behaviour change techniques to theoretical constructs. The intervention had 5 sections on Med diet and eating healthily. • Risk perception; Outcome expectancy; Self-efficacy; Planning; Action control	No control	Mediterranean Diet Adherence Screener (MEDAS)	Measures: •Dietary behaviours index •Psychological constructs 1.Positive diet-specific outcome expectancy 2.Diet specific planning 3.Diet specific action control 4.Stages of change.	The intervention showed overall improvements in Med diet adherence.

*RCT* Randomised control trial, *REP*(Reporting), *IV* (Internal validity), *EV* (External validity), *TTM* Transtheoretical Model, *N* = 9

### Methodological quality

The modified version of Downs and Blacks [42] quality checklist was used as some of the included studies were non-randomised studies. Question 27 was modified to “Did the study have sufficient power?” with one point awarded if a sample size calculation was completed [43]. Two researchers (DT&ES) independently assessed the quality of the studies. The Downs and Blacks quality checklist is considered a reliable and valid tool suitable for the use in random and non-random studies [44]. Studies were assessed on quality of reporting (10 questions; partially = 1, no = 0 or yes = 2, or yes =1, no = 0), external validity (3 questions; yes = 1, no = 0,unable to determine = 0), internal validity – measurement bias (7questions; yes = 1, no = 0, unable to determine = 0), internal validity – selection bias (6 questions; yes = 1, no = 0, unable to determine = 0) and power (1 question; yes = 1, no = 0, unable to determine = 0), equating to a total achievable score of 28 (see Table 3). Studies that scored less than 14 were considered poor, those that scored between 14 and 18 were considered fair, those that scored between 19 and 23 were considered good and those scoring between 24 and 28 were considered excellent [45].

**Table 3:** Quality checklist scores for included studies

Author	Reporting	Internal Validity (Bias)	Internal Validity (Confounding)	External validity	Power	Total
Abood, D.A et al. [33]	7	6	4	2	1	20
Manios, Y et al. [34]	8	5	2	0	0	15
Petrogianni, M et al. [35]	9	5	3	0	0	17
McPhail, M et al. [36]	11	5	5	2	0	23
Miller, C.K et al. [37]	11	6	5	2	1	25
Rodriguez, M.A et al. [38]	7	5	4	1	0	17
Peters, N.C et al. [39]	8	5	3	3	1	20
LeBlanc, V et al. [40]	10	5	3	1	1	20
Schwarzer, R et al. [41]	9	4	3	1	0	17

Quality checklist Black and Downs [35] *n* = 9

In addition to study quality being formally assessed, the Theory Coding Scheme (TCS) [26] was used to assess the extent to which theory was used to design behaviour change interventions within each study. The TCS consists of 19 items across 6 categories relating to; whether a theory was mentioned, if the relevant theoretical constructs are targeted, if theory was used to select participants and/or tailor interventions and if the relevant constructs were measured, if theory was tested and if theory was refined. Responses to all items with the exception of item 7 and 10 with a “yes” were given 1 point and those responded with a “no” and “don’t know” were given 0 points. Items 7 (All intervention techniques are explicitly linked to at least 1 theory-relevant construct) and 10 (All theory-relevant constructs are explicitly linked to at least 1 intervention technique) were given 2 points if the criteria were met (see Table 4). Similar scoring has previously been applied [46]. Similar to previous research using the TCS [47], this review scored each study as having a weak (0–7), moderate (8–15), or strong (16–23) use of theory. There was an initial 95% agreement of codes, which demonstrates an acceptable level of agreement. Discussion between researchers resolved any differences within the coding process.

**Table 4:** Assessment of Theory Application in Included Studies Using the Theory Coding Scheme (TCS)

Application	Abood [33]	Manios [34]	Petrogianni [35]	MacPhail [36]	Miller [37]	Rodriquez [38]	Peters [39]	Le Blanc [40]	Schwarzer [41]
1.Theory mentioned	1	1	1	1	1	1	1	1	1
2.Targeted construct mentioned as predictor of behaviour	1	0	0	1	0	1	1	1	1
3.Intervention based on single theory	1	0	0	1	1	1	1	1	1
4.Theory used to select recipients for the intervention	0	0	0	0	0	0	0	0	0
5. Theory used to select/develop intervention techniques	1	1	1	1	0	1	1	1	1
6. Theory used to tailor intervention techniques to recipients	0	0	0	0	0	1	0	0	0
7. All intervention techniques are explicitly linked to at least 1 theory-relevant construct	0	2	2	2	0	2	0	0	2
8. At least 1, but not all, of the intervention techniques are explicitly linked to at least 1 theory-relevant construct	1	0	0	0	1	1	1	1	0
9. Group of techniques is linked to a group of constructs	0	1	1	0	0	0	0	0	1
10. All theory-relevant constructs are explicitly linked to at least 1 intervention technique	0	2	2	2	0	2	0	0	2
11. At least 1, but not all, theory-relevant constructs are explicitly linked to at least 1 Intervention technique	1	0	0	0	1	1	0	1	0
12. Theory-relevant con0s1tructs are measured	1	0	0	1	1	1	1	1	1
13. Quality of measures	1	0	0	1	1	1	1	1	1
14. Randomization of participants to condition	1	1	1	1	1	1	1	0	0
15. Changes in measured theory-relevant constructs	1	0	1	0	1	1	0	1	0
16. Mediational analysis of constructs	0	0	0	1	1	0	0	1	1
17. Results discussed in relation to theory	1	0	1	1	1	1	1	1	1
18. Appropriate support for theory	0	0	0	0	1	0	0	1	1
19. Results used to refine theory	0	0	0	0	0	0	0	0	0
Total	11	7	8	13	11	16	9	12	14

Nineteen items of the theory coding scheme (TCS) Michie et al. [26]. 9 included studies

### Treatment fidelity

Treatment fidelity was assessed using a 29-item checklist [48] which mapped onto 5 domains identified by Bellg [28]. 1) treatment design (6 items); 2) treatment providers (7 items); 3) delivery of treatment (9 items); 4) receipt of treatment (5 items); enactment of treatment skills (2 items). The ability to draw solid conclusions from a study may be decreased, if any one of the domains lack consideration [48] (see Table 5).

**Table 5:** Fidelity of studies across 5 domains

Author	Study design	Training providers	Delivery	Receipt	Enactment	Number of components
Abood et al. [33]	✓		✓			2/5
Manios et al. [34]	✓		✓			2/5
Petrogianni et al. [35]	✓		✓	✓		3/5
MacPhail et al. [36]	✓		✓	✓		3/5
Miller et al. [37]	✓	✓	✓	✓	✓	5/5
Rodriquez et al. [38]	✓		✓	✓		3/5
Peters et al. [39]	✓		✓	✓	✓	4/5
Le Blanc et al. [40]	✓	✓	✓	✓	✓	5/5
Schwarzer et al. [41]	✓					1/5

Five domains of treatment fidelity, *n* = 9

## Results

### Study characteristic

The basic characteristics of included studies are shown in Table 2.

### Type of studies

**Fig. 1:**
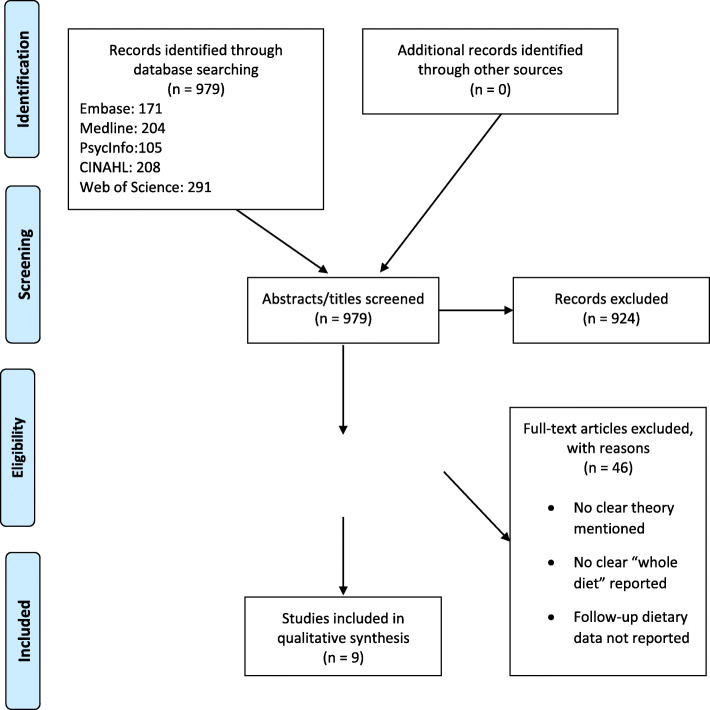
PRISMA flow chart identifying and screening studies, eligibility of studies and included studies *n* = 9

Nine studies met the inclusion criteria (see Fig. 1). Seven of the included studies were RCT’s [33–39] and 2 non-RCT’s [40, 41].

### Type of participants

In all studies, participants had a mean age ranging from 34 to 72 years. Females represented between 45 and 100% of the overall sample. One study [35] did not state the number of males and females who participated. Six of the included studies had apparent healthy participants [33, 34, 37, 39–41]. Three of the included studies had participants with a clinical diagnosis [35, 36, 38]. Of the nine studies, one was carried out in Australia [36], four in the USA [33, 37–39], one in Canada [40], and three in the Mediterranean (Greece, Italy and Spain) [34, 35, 41].

### Type of dietary pattern

All studies included a whole dietary pattern that took into consideration the main food groups: protein, grains/carbohydrates, oil and fats, dairy, fruit/vegetable (*n* = 9). Two of the nine studies specifically examined the Mediterranean diet [40, 41], and one examined the DASH diet [38].

### Type of primary outcome

Outcome measures varied across studies. Two studies used the HEI-2005 to assess overall diet quality and adherence to the recommended diet [34, 35], with higher scores representing better diet quality. One study assessed adherence to the Mediterranean diet with the Mediterranean Diet Adherence Screener (MEDAS) [41], with higher scores representing higher adherence to the Mediterranean diet. One study used the Diet Guidelines Index (DGI) to measure adherence to healthy recommendations over the previous month. A diet score is obtained with a range of 0–150, with higher scores representing higher levels of healthy eating [36]. One study assessed dietary behaviour with a food frequency questionnaire [49] and compliance to USDA Food Pyramid [33, 50]. One study used the AHEI-2010 to assess diet quality [37] with a total score between 0 and 110, with the higher score representing better diet quality. One study [40] assessed the level of adherence to the Mediterranean diet with a Medscore, which was calculated based on the food frequency questionnaire used in the study. Scores ranged from 0 to 44, with higher scores representing higher adherence to the Mediterranean diet. One study captured recommended foods by a 24-h recall questionnaire and compliance with USDA Food Pyramid [39, 50]. Finally, one study [38] used the Willett Food frequency Questionnaire [51] to derive a DASH adherence score, with a potential DASH score of 1–40 over 8 food components. Each component score between 1 and 5, with a higher score representing higher adherence.

### Quality of studies

Out of a total score of 28, all 9 included studies scored between 15 and 25 on the Black and Downs quality assessment checklist (see Table 3), with one study scoring 25 which is considered excellent quality [37]. Four studies scored between 19 and 23 which is considered good quality [33, 36, 39, 40], and the remaining four studies scoring between 14 and 18 which is considered fair quality [34, 35, 38, 41]. Overall, the 9 included studies scored high on the first subscale of the checklist (reporting). None of the included studies met the criteria for “external validity” subscale, with two studies scoring zero [34, 35]. The following section is internal validity-bias which studies scored relatively high on this subsection with scores between 4 and 6 out of a possible 7. The following subsection is internal validity-confounding (selection bias), which yielded the most variety of scores, which may be due to having different experimental designs. Only one of the RCTs [36] reported sufficiently on randomised intervention assignment concealment. Lastly, power to detect a significant effect was reported by 4 studies [33, 37, 39, 40].

### Impact of intervention on dietary behaviour

Two studies [40, 41] examined the impact of a theory-based intervention on adherence to the Mediterranean diet. Both studies calculated an overall Medscore pre-post intervention, calculated from the Mediterranean Diet Adherence Screener (MEDAS) [41], or a food frequency questionnaire [40]. Both studies reported a significant increase in Medscore post intervention. One study [38] examined the impact of a tailored behavioural intervention (TBI) on adherence to the DASH diet, compared to a non-tailored intervention (NTI) and usual care (UC) group. At 6 months follow-up, TBI had a higher DASH score than UC and NTI. However, for individual components of the DASH diet such as fruit and vegetables, and wholegrains, there was no significant difference between groups on scores at 6-month follow-up. The remaining 6 studies examined individual components of dietary behaviours based on AHEI [37], HEI [34, 35] DGI [36], FFQ [33] and 24 h recall/MyPyramid [39]^.^ From theses 6 studies, one study reported no improvement in dietary behaviour [36]. Only one study reported a significant improvement in fruit [37], vegetable intake [35], carbohydrates/grains [34] and dairy [34]. Two studies reported improvements in protein (fish, poultry, beans, meat, or eggs) [37, 39] and total fats [33, 34].

### Extent of theory use

The extent to which theory was used within the selected studies was assessed using the TCS (Table 4) [26]. From the 9 included studies, the mean total TCS score across studies was 11, which is a moderate application of theory. One study [34] showed a weak application of theory, seven studies [33, 35–37, 39–41] were moderate, and one study showed a strong application of theory [38]. These scores suggest that theory had not been extensively applied to the design, implementation, and evaluation of behaviour change interventions, and/or theory use was reported with insufficient detail. These scores suggest that most studies are not explicitly reporting theory use in sufficient detail and/or fail to rigorously apply theory to intervention design and implementation. The following section describes the use of theory within the selected studies in terms of the 6 categories of coded items of the TCS [26]: (1) mention of theory; (2) targeting of theoretical constructs;(3) using theory to select recipients or tailor interventions; (4),measurement of constructs; (5) testing of mediation effects; (6) and refining theory.

#### Category 1: mention of theory (items 1–3)

All studies (*N* = 9) mentioned a theory (item 1, Table 4), with only 6 studies referring to theory as a predictor of behaviour and provided evidence of the association of the theory or theoretical construct and target behaviour. For example, one study using the Health Belief Model [33] stated that the best predictor of nutrition related behaviour change is the benefit-cost ratio, and for a change in nutrition behaviour to occur, the perceived benefits must outweigh the barriers. Out of the 9 studies, 7 were reported to be a single theory (item 3, Table 4) such as HAPA, SDT and TTM, while 2 studies combined theories (HBM and SCT).

#### Category 2: are relevant constructs targeted (item 5, 7–11)

Eight of the studies used theory or predictors to select/develop intervention techniques (Item 5, Table 4). Regarding linking intervention techniques to theoretical constructs, only 4 studies explicitly linked all intervention techniques to at least one theoretical construct (Item 7, Table 4), with a further 5 studies linking at least one, but not all, intervention techniques to at least one theoretical construct (Item 8, Table 4). Three studies linked a group of techniques to a group of constructs (Item 9, Table 4). Only 4 studies explicitly linked all relevant theoretical constructs to at least one intervention technique (Item 10, Table 4), with a further 4 studies linking at least one, but not all, constructs with at least one technique (Item 11, Table 4). For example, one study [33] used the HBM to develop an educational intervention to improve dietary practices for CVD prevention. However, the intervention focused on perceived benefits and barriers and neglected other key concepts such as susceptibility and severity of illness, health motivation and perceived control. Another study [39] used the SCT model to develop a dietary intervention and focused their intervention techniques on self-regulation techniques, such as self-monitoring and goal setting, neglecting concepts such as outcome expectancy. Therefore, more than half (*N* = 5) of these studies did not utilise the full predictive power of their chosen theory.

#### Category 3: is theory to select participants or tailor interventions

None of the included studies used theory to select participants (Item 4, Table 4), and only 1 study tailored intervention techniques to the participants. Therefore, the intervention differed for subgroups of participants that varied for a particular construct at baseline (Item 6, Table 4). This study was based on the TTM, and the intervention delivered to each participant varied depending on their stage of change at baseline.

#### Category 4: are relevant constructs measured

Seven of the studies reported measuring theoretical constructs pre-post intervention (Item 12, Table 4), and reporting on the validity and reliability of the scales used to measure constructs/predictors (Item 13, Table 4).

#### Category 5: testing theory

Seven of the studies reported randomisation, two studies were non-RCTs (Item 14, Table 4). Four of the studies interventions changed the target theoretical constructs. For example, one study [33] using the HBM significantly increased perceived benefits of adoption of positive dietary behaviours and increased nutrition knowledge of CVD and cancer. Also, another study [52] reported that HAPA outcomes in the intervention group reported significantly greater frequency of action planning, and action and coping self-efficacy at follow-up (Item 15, Table 4). Seven of the studies discussed the results in relation to theory (Item 16, Table 4) and three provided support for theory (Item 17, Table 4). That is, studies reported that constructs within the theory, significantly mediated the relationship between the intervention and outcomes*.* For example, one study [53] that used self-determination theory found that eating related self-determined motivation was associated with an increased adherence to the Mediterranean diet.

#### Category 6: refining theory

Refining of theory, or suggestions for future refinement was not reported by any of the included studies (Item 18, Table 4).

### Fidelity of interventions

Of the 9 included studies, two studies included an assessment on all 5 domains [37, 40]. One study included an assessment on only one domain [41]. Two studies included an assessment on two domains [33, 34]. Three studies included an assessment on three domains [35, 36, 38]. One study included an assessment on four domains [39] (see Table 5).

#### Study design

All studies made an assessment on study design [33–41], with information about treatment dose provided in the intervention condition, and two providing information on treatment dose in the comparison group [37, 39]. All studies reported underpinning theory [33–41]. No further trained providers were employed to allow for setbacks.

#### Training providers

Two studies provided information on training providers [37, 40]. These studies provided information on how trainers were trained and standardisation of provider training. Strategies to enhance training providers included, using the same provider throughout the intervention [40], use of certified trainers [40], and train all providers together [37].

#### Delivery of treatment

Eight of the studies made at least one assessment on the delivery of treatment [33–40], which was assessed through direct observation of the intervention. Making sure that the interventions were delivered, and the appropriate dose given, being the most reported item in this domain.

Various criteria were used to evaluate the treatment delivery. For example, one study [39] used a checklist after each session to measure degree of adherence, and class attendance [34, 39]. In another study, participants reported on the acceptability of the intervention [36], and how the participants rated the overall delivery of the intervention [40]. Other strategies used to assess delivery of treatment were the use of manuals to aid delivery [33, 36–38].

#### Receipt of treatment

Six studies made at least one assessment on the receipt of treatment. Various strategies were used to assess receipt between authors and included ensuring that participants understood the intervention [35–40] and providing resources to enable participants to perform the behaviour [39, 40]. Other strategies to assess receipt of treatment included reviewing self-monitoring data [35, 37], and assessing confidence in behavioural skills [36–39].

#### Enactment of treatment skills

Observation and practice of skills required within interventions were included in three of the studies. Observation of these skills in daily life were carried out in two of the studies [39, 41]. Other strategies to assess whether treatment was being enacted were daily self-monitoring and tracking devices [37].

## Discussion

To our knowledge, this is the first systematic review to assess the effectiveness of dietary interventions promoting a whole dietary pattern using a social cognition model. This systematic review has investigated the extent of SCM use in designing interventions to increase adherence to whole dietary patterns and explored the associations between theory use and intervention effectiveness. This review also explored the extent to which the 5 domains of treatment fidelity are reported in the selected studies. We found that the overall scores, across the 9 included studies, measured by the TCS averaged 10 out of a possible 21 points. This suggests that the studies were not explicitly theoretically informed or used to their full extent, even though theory was explicitly mentioned. This review also found that only two studies made at least one assessment on all five fidelity domains. As all five components of fidelity are mutually exclusive. The validity of a study is potentially compromised with inattention to any one of the 5 fidelity domains [48].

Five behaviour change theories were used in the studies of the current review (HAPA, HBM, SCT, SDT, TTM), with HAPA used by 3 of the 9 included studies. Out of the 9 studies, one study [36] showed no improvements in diet following the intervention based on the Diet Guidelines Index (DGI) to create an overall single score of diet quality. Previous research has stated that the way in which dietary scales score individual food groups to create a single score can be problematic [54], as observed associations could be due to single components rather than the overall dietary pattern [7]. Small-scale scores are less informative, as the extremes and the inherent characteristics of a pattern or a behaviour may not be fully captured [7]. Furthermore, research has shown that participants had better control of their diet and ate more healthily compared to the general population and therefore, changes in diet quality could not be detected [36]. Also, those in the intervention group perceived less risk awareness to those in the control group, which could have affected their engagement in the intervention [36]. Awareness of the importance of balanced nutrition is shown to be an important factor that may influence dietary choices [55, 56].

Five of the studies used a dietary scale that reported individual food group scores. All five studies improved dietary scores for at least one food group. One study found a significant improvement in fruit intake [37], vegetable intake [35], carbohydrates/grains [34], and dairy [34]. Two studies reported improvements in protein (fish, poultry, beans, meat, or eggs) [37, 39], and total fats [33, 34]. These findings are consistent with a previous review which found that out of half the studies examined, at least one aspect of diet had not improved, with a further 5 studies showing no improvement in diet quality. However, in the same review, one quarter of the studies were found to be explicitly theoretically informed (based on the Theory Coding Scheme), and significantly improved diet quality. Of these 10 studies, 8 reported improvements in fruit and vegetables [25] suggesting that interventions that use behaviour change theory rigorously, lead to better outcomes in trials.

The current review found limited association between the use of psychological theory and improved intervention outcomes, with only three of the studies in this review reporting an association between theory and intervention effectiveness (assessed through individual TCS items). One possible explanation for the relatively limited effectiveness of the interventions reviewed in the present review is that they apply theory insufficiently. The current review showed that the included studies revealed theoretical implementation weaknesses. Most notably, linking all BCTs to theoretical constructs were met by only 4 out of the 9 studies. Compared to previous findings [21, 57], this review observed a closer link between intervention and theory, measured by a higher percentage of studies reporting on linkage between theoretical constructs and intervention techniques (TCS items 7–11). However, in the current review, only studies that explicitly mentioned theory were included. Previous research targeted interventions whether theory was mentioned or not for the target behaviour, with only half the studies reported to be explicitly based on theory, and of those, few targeted all theoretical constructs or linked all BCTs to theoretical constructs [21].

Theory based interventions can help us understand processes and effectiveness of interventions [26] by identifying key constructs that are shown to be related to behaviour and behaviour change techniques related to the relevant constructs, that can be used as a target for intervention design. Research has found that interventions tailored on theoretical concepts were more effective than those tailored on behaviour alone [58]. However, as more than half of the included studies in the current review did not report on this concept fully, the findings limit the extent of evidence of behaviour change factors [59]. Overall, these finding highlight the need for clearer selection, application, and reporting of theory use in the design, implementation, and evaluation of dietary intervention.

Linking BCT’s to theory provides an opportunity to refine theory [26] and while the current review found that most of the studies linked at least one BCT to theoretical constructs, none of the studies used the results to refine theory. It is important to address this, as not only is theory important in the developmental stages of intervention design and future interventions, but to the advancement of our understanding of how interventions affect behaviour. This lack of refining theory from interventions is common, with similar results found in recent research [59–61].

A second explanation to the relatively limited effectiveness of the interventions reviewed in the present review is that the interventions may not have been delivered as the designers intended. This cannot be ruled out, as treatment fidelity was poorly reported in the current review studies. According to Borrelli [48]. there are five domains of treatment fidelity: study design, training, delivery, receipt, and enactment, all of which are mutually exclusive. The validity of a study is potentially compromised with inattention to any one of the 5 fidelity domains. The overall reporting of treatment fidelity in the current review is poor, with only 3 studies reporting on more than three of the five domains. This finding is similar to other reviews considering fidelity [48, 62]. Overall, we found that regardless of the theory coding scheme score, those studies that reported high on fidelity, reported improvements in more food groups than those with lower fidelity. For example, one study [38] that scored the highest in the theory coding scheme but low on fidelity, reported a significant improvement in overall DASH score, but not in any of the individual food groups. Furthermore, two of the included studies that scored relatively low on the theory coding scheme and high on fidelity, reported better adherence to the Mediterranean diet [40], and improvement to several of the food groups including fruit, red meat, processed meat and total AHEI scores [37]. Moreover, two studies scoring the lowest on fidelity [33, 34], reported improvements on less food groups, which did not include fruit or vegetables. However, these two studies also scored relatively low on the TCS. This finding demonstrates that, while the TCS addresses fidelity of treatment such as, explicitly identifying and use of theory as a basis for intervention design, there are other factors that are not addressed. For example, if insignificant results were found in an intervention and only one or two of the domains were of high fidelity, it is possible that the insignificant results were due to a lack of attention in the other domains [27], such as the training providers may not have been adequately trained. Therefore, in order to enhance the transition from theory to practice, we recommend that intervention designers include a plan to assess and monitor treatment fidelity based on the 5 domains proposed by Borrelli [48].

Using theory to design behaviour change interventions have been criticised, as they specify what theoretical constructs (i.e. intentions) should be changed to change behaviour, but do not specify how constructs can be changed. However, systematic reviews have recently started to identify links between theoretical constructs and BCTs, enhancing the effectiveness of behaviour change interventions [63]. It has been suggested, those that target change mechanisms at population, community and individual levels are the most effective [64], suggesting that behaviour change interventions may benefit from drawing on a wider range of theories than Social Cognition Models [20]. Recently, new approaches to behaviour change, and the implementation and evaluation of interventions has been developed, in particular, the Behaviour Change Wheel, COM-B model and the BCT taxonomy which helps build the bridge between predicting behaviour and actual behaviour, by specifying the “active ingredients” of the intervention, and this classification will facilitate replication of interventions [65]. The Behaviour Change Wheel seeks to provide a framework, that other theories can be considered. Social Cognition Models constructs mainly fall into the reflective motivation component of the COM-B model and either minimally or not at all into the other 5 components [20]. The COM-B model is a holistic approach for changing behaviour, based on a model of an individual, rather than a mechanistic process of identifying determinants of behaviour based on factors accounting for variation in current behaviour between individuals [20]. The BCW incorporates the COM-B model, TDF and BCT’s in a systematic approach in designing an intervention. The BCW is gaining popularity in developing interventions in a range of health behaviours including dietary behaviour [66, 67]. Therefore, more research is needed, using new approaches to understand dietary behaviour, and in the development and evaluation of complex interventions [68].

### Strengths and limitations

A major strength of the current review is the use of the TCS, which allowed for a deeper exploration of the extent of psychological theory driven interventions, and also our understanding of shortcomings in the reporting and implementation on the use of psychological theory. This review did not conduct a meta-analysis, however, the differences found in the included studies populations, interventions and behavioural theories would make the average effect across studies difficult to interpret [69]. Relevant studies may have been excluded due to selection criteria and search terms. For example, studies that are not in English but used theory and relevant to this review would be missed and studies that failed to report they used a behaviour change theory. However, full articles were obtained for possible inclusion for potentially relevant articles, even if theory was not explicitly mentioned in the abstract, further minimising potential bias. Coding of the TCS may be subject to misclassification bias, however, two researchers (DT&LS) interpreted and coded the TCS items to reduce any bias.

## Conclusion

To our knowledge this is the first review to examine psychological theory driven interventions that use a whole dietary pattern. We have found that, while all the included studies mentioned theory, total scores were mostly moderate, suggesting that theory had not been extensively applied to the design, implementation and evaluation of behaviour change interventions, and/or theory use was reported with insufficient detail. We recommend that future interventions explicitly link theory and outcome, to allow identification of the most salient intervention techniques and behaviour change theory, to advance our understanding of behaviour change. To enhance the transition from theory to practice, we recommend researchers use a fidelity framework to guide the reporting of treatment fidelity in future research. Mixed results were observed for the effectiveness of theory-based interventions. With the small number of included studies, only one of which was high quality, findings should be interpreted with caution. Future reviews should include both theory and non-theory interventions, to provide evidence of the effectiveness of psychological based interventions compared to no theory use.

## Supplementary Information

**Additional file 1.** PRISMA 2009 checklist. The PRISMA 2009 checklist is a 27 item checklist for the reporting of a systematic review and/or meta-analysis, which include the title, abstract, methods, results, discussion, and funding.

## Data Availability

All relevant data is included within the manuscript file.

## Abbreviations

BCC: Behaviour change consortium
BCT: Behaviour change techniques
COM-B: Capability opportunity, motivation and behaviour
CG: Control group
DASH: Dietary Approaches to Stop Hypertension
DGI: Dietary guidelines index
EPOC: Cochrane effective practice and organisation of care
HAPA: Health action process approach
HBM: Health belief model
HEI: Healthy eating index
IG: Intervention group
MEDAS: Mediterranean diet adherence screener
MIND: Mediterranean intervention for neurodegenerative delay
NCD: Non-communicable disease
PICOS: Population, intervention, comparison, outcome, study design
PRISMA: Preferred reporting items for systematic reviews and meta-analysis
PROSPERO: International prospective register of systematic reviews
RCT: Randomised controlled trial
SCT: Social cognitive theory
SDT: Social determination theory
TPB: Theory planned behaviour
TCS: Theory coding scheme
TTM: Transtheoretical model
USA: United states of America
USDA: Dietary guidelines for Americans
WHO: World health organisation

## References

[1] World Health Organization. World health statistics 2016: monitoring health for the SDGs sustainable development goals. World Health Organization. Available from: https://www.who.int/news-room/fact-sheets/detail/noncommunicable-diseases.

[2] Murray CJ (2019). Health effects of dietary risks in 195 countries, 1990–2017: a systematic analysis for the global burden of disease study. Lancet..

[3] Yang Q, Zhang Z, Gregg EW, Flanders WD, Merritt R, Hu FB (2014). Added sugar intake and cardiovascular diseases mortality among US adults. JAM Intern Med.

[4] Tapsell LC, Neale EP, Satija A, Hu FB (2016). Foods, nutrients, and dietary patterns: interconnections and implications for dietary guidelines. Adv Nutr.

[5] Willett W (2013). Nutritional epidemiology.

[6] Mumme KD, von Hurst PR, Conlon CA, Jones B, Haskell-Ramsay CF, Stonehouse W, Heath AL, Coad J, Beck KL (2019). Study protocol: associations between dietary patterns, cognitive function and metabolic syndrome in older adults–a cross-sectional study. BMC Public Health.

[7] Schulze MB, Martínez-González MA, Fung TT, Lichtenstein AH, Forouhi NG (2018). Food based dietary patterns and chronic disease prevention. BMJ..

[8] Imamura F, Micha R, Khatibzadeh S (2015). Dietary quality among men and women in 187 countries in 1990 and 2010: a systematic assessment. Lancet Glob Health.

[9] Choices NH. The eatwell plate. 2015. URL: http://www.nhs.uk/Livewell/Goodfood/Pages/eatwell-plate.aspx [accessed 2015-01-24] [WebCite Cache ID 6Vo8KHOM4].

[10] MyPlate C. tips for to a great plate. DG TipSheet No. 1, USDA. Center for Nutrition Policy and Promotion. June. 2011.

[11] Panagiotakos DB, Pitsavos C, Arvaniti F, Stefanadis C. Adherence to the Mediterranean food pattern predicts the prevalence of hypertension, hypercholesterolemia, diabetes and obesity, among healthy adults; the accuracy of the MedDietScore. Prev Med. 2007;44(4):335–40. 10.1016/j.ypmed.2006.12.009 17350085

[12] Webb T, Joseph J, Yardley L, Michie S (2010). Using the internet to promote health behavior change: a systematic review and meta-analysis of the impact of theoretical basis, use of behavior change techniques, and mode of delivery on efficacy. JMIR..

[13] Prestwich A, Webb TL, Connor M (2015). Using theory to develop and test interventions to promote changes in health behaviour: evidence, issues, and recommendations. Curr Opin Psychol.

[14] Ajzen I (1991). The theory of planned behavior. Organizational behavior and human decision processes..

[15] Coulson NS, Ferguson MA, Henshaw H, Heffernan E (2016). Applying theories of health behaviour and change to hearing health research: Time for a new approach. Int J Audiol.

[16] Conner M, Norman P. Predicting and changing health behaviour: research and practice with social cognition models. Maidenhead. 2015.

[17] McDermott MS, Oliver M, Simnadis T (2015). The theory of planned behaviour and dietary patterns: a systematic review and meta-analysis. Prev Med.

[18] Omondi DO, Walingo MK, Mbagaya GM, Othuon LOA (2011). Predicting dietary practice behavior among type 2 diabetics using the theory of planned behavior and mixed methods design. World Acad Sci Eng Technol.

[19] Morris MC, Tangney CC, Wang Y, Sacks FM, Bennett DA, Aggarwal NT (2015). MIND diet associated with reduced incidence of Alzheimer's disease. Alzheimers Dement.

[20] Sacks FM, Moore TJ, Appel LJ, Obarzanek E, Cutler JA, Vollmer WM, Vogt TM, Karanja N, Svetkey LP, Lin PH, Bray GA. A dietary approach to prevent hypertension: a review of the Dietary Approaches to Stop Hypertension (DASH) Study. Clin Cardiol. 22(S3):6–10.10.1002/clc.496022150310410299

[21] Prestwich A, Sniehotta FF, Whittingham C, Dombrowski SU, Rogers L, Michie S (2014). Does theory influence the effectiveness of health behavior interventions? Meta-analysis. Health Psychol..

[22] Diep CS, Chen TA, Davies VF, Baranowski JC, Baranowski T (2014). Influence of behavioral theory on fruit and vegetable intervention effectiveness among children: a meta-analysis. J Nutr Educ Behav.

[23] Gardner B, Wardle J, Poston L, Croker H (2011). Changing diet and physical activity to reduce gestational weight gain: a meta-analysis. Obes Rev.

[24] Hagger MS, Cameron LD, Hamilton K, Hankonen N, Lintunen T, editors. The handbook of behavior change. Cambridge University Press; 2020.

[25] Avery KN, Donovan JL, Horwood J, Lane JA (2013). Behavior theory for dietary interventions for cancer prevention: a systematic review of utilization and effectiveness in creating behavior change. Cancer Causes Control.

[26] Michie S, Prestwich A (2010). Are interventions theory-based? Development of a theory coding scheme. Health Psychol.

[27] O’Shea O, McCormick R, Bradley JM, O’Neill B (2016). Fidelity review: a scoping review of the methods used to evaluate treatment fidelity in behavioural change interventions. Phys Ther Rev.

[28] Bellg A, Borrelli B, Resnick B, Hecht J, Minicucci D, Ory M (2004). Enhancing treatment fidelity in health behavior change studies: best practices and recommendations from the NIH behavior change consortium. Health Psychol.

[29] Broers VJ, De Breucker C, Van den Broucke S, Luminet O (2017). A systematic review and meta-analysis of the effectiveness of nudging to increase fruit and vegetable choice. Eur J Public Health.

[30] Al Rawahi SH, Asimakopoulou K, Newton JT (2018). Factors related to reducing free sugar intake among white ethnic adults in the UK: a qualitative study. BDJ Open.

[31] Stacey FG, James EL, Chapman K, Courneya KS, Lubans DR (2015). A systematic review and meta-analysis of social cognitive theory-based physical activity and/or nutrition behavior change interventions for cancer survivors. J Cancer Surviv.

[32] Moher, D., Liberati, A., Tetzlaff, J., Altman, D. G., & Prisma Group. Preferred reporting items for systematic reviews and meta-analyses: the PRISMA statement. PLoS med 2009; 6(7), e1000097. 10.1371/journal.pmed.1000097 PMC2707599 19621072

[33] Abood DA, Black DR, Feral D (2003). Nutrition education worksite intervention for university staff: application of the health belief model. J Nutri Educ Behav.

[34] Manios Y, Moschonis G, Katsaroli I, Grammatikaki E, Tanagra S (2007). Changes in diet quality score, macro-and micronutrients intake following a nutrition education intervention in postmenopausal women. J Hum Nutri Diet.

[35] Petrogianni M, Kanellakis S, Kallianioti K, Argyropoulou D, Pitsavos C, Manios Y (2013). A multicomponent lifestyle intervention produces favourable changes in diet quality and cardiometabolic risk indices in hypercholesterolaemic adults. J Hum Nutri Diet..

[36] MacPhail M, Mullan B, Sharpe L, MacCan C, Todd J (2014). Using the health action process approach to predict and improve health outcomes in individuals with type 2 diabetes mellitus. Diabetes Metab Syndr Obes.

[37] Miller CK, Weinhold KR, Nagaraja HN (2016). Impact of a worksite diabetes prevention intervention on diet quality and social cognitive influences of health behavior: a randomized controlled trial. J Nutri Educ Behav..

[38] Rodriguez MA, Friedberg JP, DiGiovanni A, Wang B, Wylie-Rosett J, Hyoung S, Natarajan S (2019). A tailored behavioral intervention to promote adherence to the DASH diet. Am J Health Behav.

[39] Peters NC, Contento IR, Kronenberg F, Coleton M (2014). Adherence in a 1-year whole foods eating pattern intervention with healthy postmenopausal women. Public Health Nutri.

[40] Leblanc V, Bégin C, Hudon AM, Royer MM, Corneau L, Dodin S, Lemieux S (2015). Effects of a nutritional intervention program based on the self-determination theory and promoting the Mediterranean diet. Health psychology open.

[41] Schwarzer R, Fleig L, Warner LM, Gholami M, Serra-Majem L, Ngo J, ... & Giannakis G. Who benefits from a dietary online intervention? Evidence from Italy, Spain and Greece. Public Health Nutri. 2017; 20(5): 938–947. 10.1017/S1368980016002913 PMC10261616 27829475

[42] Downs SH, Black N (1998). The feasibility of creating a checklist for the assessment of the methodological quality both of randomised and non-randomised studies of health care interventions. J Epidemiol Community Health.

[43] Morton S, Barton CJ, Rice S, Morrissey D (2014). Risk factors and successful interventions for cricket-related low back pain: a systematic review. Br J Sports Med.

[44] Saunders LD, Mustafa Soomro G, Buckingham J, Jamtvedt G, Raina P (2003). Assessing the methodological quality of nonrandomised intervention studies. West J Nurs Res.

[45] O’Connor SR, Tully MA, Ryan B, Bradley JM, Baxter GD, McDonough SM (2015). Failure of a numerical quality assessment scale to identify potential risk of bias in a systematic review: a comparison study. BMC research notes.

[46] Alageel S, Guilford MC, Wright AJ. Multiple health behaviour change interventions for primary prevention of cardiovascular disease in primary care: systematic review and meta-analysis. BMJ open.2017;7(6): e015375. 10.1136/bmjopen-2016-015375 PMC5734412 28619779

[47] Willmott T, Pang B, Rundle-Thiele S, Badejo A (2019). Reported theory use in electronic health weight management interventions targeting young adults: a systematic review. Health Psychol Rev.

[48] Borrelli B (2011). The assessment, monitoring, and enhancement of treatment fidelity in public health clinical trials. J Public Health Dent.

[49] Boeckner LS, Kohn H, Rockwell K (1990). A risk-reduction course for adults. J Am Diet Assoc.

[50] Pyramid FG. A Guide to Daily Food Choices. Washington, DC: US Dept of Agriculture. Human Nutrition Information Service. 1992; 341–342.

[51] Willett WC, Sampson L, Stampfer MJ, Rosner B, Bain C, Witschi J, Hennekens CH, Speizer FE (1985). Reproducibility and validity of a semiquantitative food frequency questionnaire. Am J Epidemiol.

[52] Toomey E, Hardeman W, Hankonen N, Byrne M, McSharry J, Matvienko-Sikar K, Lorencatto F (2020). Focusing on fidelity: narrative review and recommendations for improving intervention fidelity within trials of health behaviour change interventions. Health Psychol Behavioral Med.

[53] Rosenstock (1966). Why people use health services. Milbank Memorial Fund Quart.

[54] Arvanti F, Panagiotakos DB (2008). Healthy indexes in public health practice and research: a review. Crit Rev Food Sci Nutr.

[55] Alkerwi A, Sauvageot N, Malan L, Shivappa N, Hébert JR (2015). Association between nutritional awareness and diet quality: evidence from the observation of cardiovascular risk factors in Luxembourg (ORISCAV-LUX) study. Nutrients..

[56] Paquette MC (2005). Perceptions of healthy eating: state of knowledge and research gaps. Can J Public Health.

[57] Demmelmaier I, Iverson MD (2018). How are behavioral theories used in interventions to promote physical activity in rheumatoid arthritis? A systematic review. Arthritis Care Res (Hoboken).

[58] Noar SM, Benac CN, Harris MS (2007). Does tailoring matter? Meta-analytic review of tailored print health behavior change interventions. Psychol Bull.

[59] Lippke S, Ziegelmann JP (2008). Theory-based health behavior change: developing, testing, and applying theories for evidence-based interventions. Appl Psychol.

[60] Baron JS, Sullivan KJ, Swaine JM (2018). Self-management interventions for skin care in people with a spinal cord injury: part 2—a systematic review of use of theory and quality of intervention reporting. Spinal Cord..

[61] Casey B, Coote S, Hayes S, Gallagher S (2018). Changing physical activity behavior in people with multiple sclerosis: a systematic review and meta-analysis. Arch Phys Med Rehabil.

[62] JaKa MM, Haapala JL, Trapl ES, Kunin-Batson AS, Olson-Bullis BA, Heerman WJ, Berge JM, Moore SM, Matheson D, Sherwood NE (2016). Reporting of treatment fidelity in behavioural paediatric obesity intervention trials: a systematic review. Obes Rev.

[63] Prestwich A, Kellar I, Conner M, Lawton R, Gardner P, Turgut L (2016). Does changing social influence engender changes in alcohol intake? A meta-analysis. J Consulting Clin Psychol.

[64] National Institiute of Health Care Excellence. Behaviour change: General approaches. Retrieved from http://www.nice.org.uk/guidance/ph6/chapter/3-recommendations.

[65] Michie S, Atkins L, West R (2014). The behaviour change wheel: a guide to designing interventions. Needed: Physician Leaders.

[66] Costello N, McKenna J, Sutton L, Deighton K, Jones B (2018). Using contemporary behavior change science to design and implement an effective nutritional intervention within professional rugby league. Int J Sport Nutrition Exercise Metabolism.

[67] McEvoy CT, Moore SE, Appleton KM, Cupples ME, Erwin C, Kee F, Prior L, Young IS, McKinley MC, Woodside JV (2018). Development of a peer support intervention to encourage dietary behaviour change towards a Mediterranean diet in adults at high cardiovascular risk. BMC Public Health.

[68] Craig P, Dieppe P, Macintyre S, Michie S, Nazareth I, Petticrew M (2008). Developing and evaluating complex interventions: the new Medical Research Council guidance. BMJ..

[69] Cochrane Collaboration. (2011). 9.1. 4 When not to use Meta-analysis in a review. Cochrane Handbook for Systematic Reviews of Interventions, 5(0).

